# Nanotechnology-Based Cancer Vaccines: Translational Barriers and Emerging Strategies

**DOI:** 10.3390/vaccines14060463

**Published:** 2026-05-22

**Authors:** Muneera Anwer, Rifat Rahman

**Affiliations:** School of Pharmacy and Medical Sciences, Griffith University, Gold Coast, QLD 4215, Australia

**Keywords:** nanotechnology, cancer vaccines, nanoparticle delivery systems, personalized nanovaccines, mRNA vaccines, translational barriers, combination immunotherapy

## Abstract

Cancer vaccines represent a promising strategy in cancer immunotherapy by inducing tumour-specific immune responses. However, their clinical efficacy remains limited due to challenges in antigen selection, including the distinction between self and non-self-antigens, as well as issues related to antigen delivery, immune activation, and tumour immune evasion. Advances in nanotechnology have introduced innovative approaches to improve vaccine stability, targeted delivery, and immunogenicity. Nanoparticle-based platforms, including lipid, polymeric, inorganic nanoparticles, and virus-like particles, enable efficient delivery of tumour antigens and immunostimulatory adjuvants to antigen-presenting cells, thereby enhancing adaptive immune responses. Despite these advances, several translational challenges persist, including immunosuppressive tumour microenvironments, inefficient lymph node targeting, safety concerns, and manufacturing limitations. This review summarizes key nanoparticle platforms used in cancer vaccine development and discusses major barriers to their clinical translation. We also emphasize platform-selection criteria, cargo-dependent carrier design, nanoparticle size constraints, engineering strategies used to improve cytosolic delivery and endosomal escape, and the current clinical pipeline of cancer nanovaccines. Additionally, emerging strategies such as personalized nanovaccines, mRNA vaccine platforms, and combination immunotherapies are highlighted as promising approaches to improve therapeutic efficacy. These advances are expected to accelerate the clinical translation of nanotechnology-enabled cancer vaccines and support the development of next-generation cancer immunotherapies.

## 1. Introduction

Cancer continues to be a leading cause of global morbidity and mortality despite advances in surgery, chemotherapy, and radiotherapy. Traditional approaches often suffer from systemic toxicity, limited specificity, and resistance, which has driven major interest in immunotherapy for cancer treatment. Among immunotherapeutic strategies, cancer vaccines aim to harness the adaptive immune system by presenting tumour-associated antigens (TAAs) or neoantigens to antigen-presenting cells (APCs), thereby eliciting cytotoxic T-lymphocyte responses that can recognize and eliminate malignant cells.

Cancer vaccine efficacy is critically dependent on the selection of appropriate tumour antigens and the use of effective immunostimulatory adjuvants. Tumour antigens are broadly categorized into tumour-associated antigens (TAAs), which are overexpressed or aberrantly expressed self-proteins, and tumour-specific antigens (TSAs), which are exclusively expressed in tumour cells [1]. Among TSAs, neoantigens derived from tumour-specific somatic mutations have gained significant attention due to their high immunogenicity and lack of central immune tolerance [2]. However, antigen selection remains a major challenge, as TAAs often induce weak immune responses due to immune tolerance, whereas TSA/neoantigen-based approaches require individualized identification, validation, and manufacturing, limiting their widespread clinical applicability [3]. In parallel, adjuvants play a crucial role in enhancing vaccine efficacy by activating innate immune pathways and promoting antigen presentation. Commonly used adjuvants in cancer vaccines include Toll-like receptor (TLR) agonists, cytokines, and stimulator of interferon genes (STING) pathway activators, which enhance dendritic cell maturation and T-cell priming [4]. Therefore, the combined optimization of antigen selection and adjuvant design is essential for achieving effective and durable anti-tumour immune responses. However, clinical efficacy has generally been modest, largely due to challenges in achieving efficient delivery, antigen stability, and robust immune activation in vivo. Review evidence highlights that cancer vaccines often fail to elicit strong T cell responses due to inefficient antigen delivery, including rapid antigen degradation, poor targeting to antigen-presenting cells, suboptimal lymphatic trafficking, and inefficient cellular uptake, together with the immunosuppressive nature of the tumour microenvironment (TME) [5]. To address these limitations, nanotechnology has emerged as a transformative solution to these limitations by enabling the design of nanoparticle-based carriers that protect antigens and adjuvants, improve lymphatic transport, and enhance uptake by APCs. Nanovaccines include platforms such as lipid nanoparticles, polymeric nanoparticles, inorganic carriers, and virus-like particles that can co-deliver antigens with immunostimulatory molecules to orchestrate more potent and durable immune responses. Evidence shows that nanovaccines improve antigen stability, increase dendritic cell uptake, and enhance antigen cross-presentation, which are the key steps for effective T cell priming and anti-tumour immunity [6,7]. The success of lipid nanoparticle (LNP) based mRNA vaccines against SARS-CoV-2 has further encouraged the interest in applying similar platforms to cancer vaccines, demonstrating that nanocarriers can facilitate efficient in vivo delivery, immune activation, and scalable manufacturing [8]. Moreover, emerging reviews have emphasized the adaptability of nanotechnology for personalized vaccine design and combination with other immunotherapies [9]. Despite these advances, significant translational barriers remain, including navigating the complex tumour microenvironment, ensuring safety and biocompatibility, and resolving manufacturing and regulatory challenges. Addressing these hurdles is critical for realizing the full clinical potential of cancer nanovaccines and forms the focus of the current mini review.

**Figure 1:**
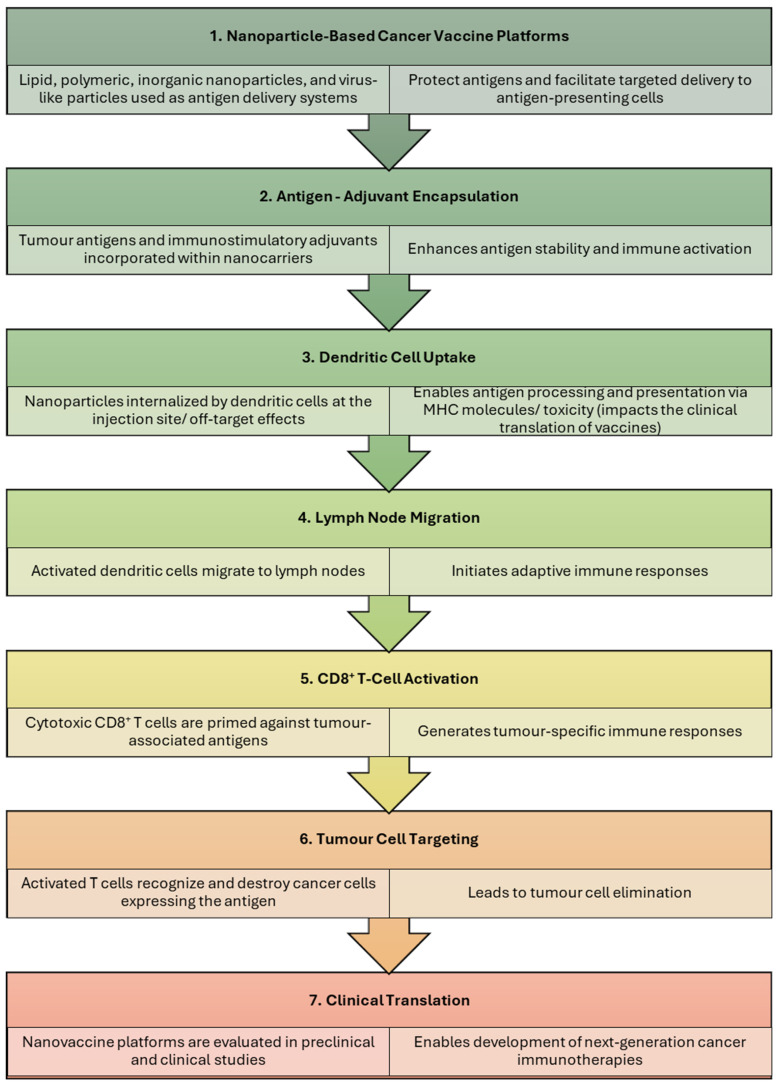
Mechanistic workflow and translational considerations of nanoparticle-based cancer vaccines. The steps involved are numbered from 1–7; the left panel represents the key process in the step, and the right panel represents the outcome. Nanoparticle platforms, including lipid, polymeric, inorganic nanoparticles, and virus-like particles, are used to deliver tumour antigens and immunostimulatory adjuvants to antigen-presenting cells. Following uptake by dendritic cells, antigens are processed and presented via major histocompatibility complex molecules, leading to T-cell activation in lymph nodes. Activated cytotoxic CD8^+^ T cells subsequently migrate to tumour sites and mediate tumour cell elimination. In addition to this therapeutic pathway, nanovaccine performance is influenced by tumour targeting efficiency, potential off-target biodistribution, and safety considerations such as cytotoxicity. These factors collectively impact the clinical translation of nanotechnology-based cancer vaccines.

Nanotechnology-based cancer vaccines enhance immune responses through a coordinated sequence of events that extend beyond conventional vaccine mechanisms. Following administration, nanoparticle carriers protect tumour antigens and adjuvants from degradation and facilitate their targeted uptake by dendritic cells, which are critical for antigen presentation. Within these antigen-presenting cells, nanoparticle-delivered antigens are processed and presented via major histocompatibility complex (MHC) class I and II pathways, enabling efficient cross-presentation and activation of cytotoxic CD8^+^ T cells. Importantly, nanocarriers can be engineered to improve lymphatic drainage and lymph node accumulation, thereby enhancing T-cell priming. In the context of cancer, this process is particularly significant because effective anti-tumour immunity requires not only antigen presentation but also overcoming tumour-induced immune suppression and generating durable effector responses capable of infiltrating tumour tissues [10,11]. The mechanistic basis of nanoparticle-enabled cancer vaccines is summarized in Figure 1.

## 2. Nanoparticle Platforms in Cancer Vaccines

A range of nanoparticle platforms has been developed to improve antigen delivery and immune activation in cancer vaccines, each offering distinct physicochemical and immunological advantages. These platforms differ in composition, payload compatibility, and capacity to modulate antigen presentation and immune responses. Several major classes of nanocarriers have been developed for cancer vaccine applications, including lipid-based, polymeric, inorganic, and virus-like particle systems. Lipid nanoparticles have emerged as one of the most clinically advanced vaccine platforms due to their biocompatibility, modular formulation, and ability to encapsulate nucleic acids and protein antigens [10,11]. LNPs are composed of ionizable lipids, helper lipids, and stabilizers that self-assemble into vesicular structures capable of protecting sensitive payloads and enhancing cellular uptake. Their success in mRNA vaccine delivery has been demonstrated most prominently in infectious disease settings and is now being actively translated into cancer vaccines, where antigen-encoding mRNA encapsulated within LNPs promotes efficient expression in antigen-presenting cells and robust T-cell activation [8]. In cancer vaccine applications, LNPs enable efficient in vivo expression of tumour antigens in antigen-presenting cells and promote strong CD8^+^ T-cell responses [12]. Polymeric nanoparticles constitute a versatile class of carriers made from biodegradable polymers such as Poly(lactic-co-glycolic acid) (PLGA), Poly(lactic acid) (PLA), and other synthetic or natural polymers. These systems can encapsulate or adsorb antigenic proteins, peptides, or nucleic acids and have been widely explored for co-delivery with adjuvants to improve immune responses. Polymeric nanovaccines offer controlled release, modifiable physicochemical properties, and the potential for surface functionalization with targeting ligands to improve uptake by dendritic cells and sustain immune stimulation over time. Recent reviews highlight their ability to enhance antigen presentation and immunogenicity when compared to soluble antigens alone [13]. Inorganic nanoparticles, including gold, silica, and metal–organic framework (MOF)-based systems, offer unique material properties that can be harnessed for cancer vaccine delivery and immune modulation. Inorganic platforms can act as rigid scaffolds for high antigen loading, intrinsic adjuvants, or as imaging contrast agents combined with vaccine payloads, enabling their applications. Their large surface area and facile surface chemistry allow multivalent antigen presentation, though careful evaluation of long-term biocompatibility and clearance is essential for clinical translation [14]. Virus-like particles (VLPs) are self-assembling protein nanostructures that resemble native viruses but lack genetic material, rendering them non-infectious while retaining high immunogenicity. VLPs provide repetitive, multivalent display of antigens, which facilitates strong B- and T-cell responses and efficient trafficking to lymphoid tissues. Their structural mimicry of viruses promotes uptake by APCs and potent adaptive immune stimulation without the safety risks associated with live viruses.

In practice, platform selection is determined by three interrelated considerations: (i) the biochemical nature of the payload, (ii) the desired release profile and intracellular fate, and (iii) the intended immunological mechanism. Nucleic acid payloads, particularly mRNA, generally favour LNPs because ionizable lipids enable high encapsulation efficiency and endosomal escape after cellular uptake. In contrast, peptide or protein antigens may be more compatible with polymeric or VLP-based platforms when the goal is prolonged antigen retention, depot formation, or multivalent display. Hydrophilic nucleic acids often require electrostatic condensation or encapsulation, whereas hydrophobic adjuvants can be incorporated into lipid bilayers or polymer matrices. Likewise, fragile cargoes benefit from platforms that minimize shear stress and enzymatic degradation during formulation and after administration. Release kinetics also inform carrier choice. Rapid cytosolic release is especially important for mRNA vaccines and for cross-presentation of peptide antigens on MHC class I molecules, favouring ionizable lipid systems or pH-responsive polymers. By contrast, when sustained antigen exposure and prolonged APC stimulation are desired, biodegradable polymers such as PLGA may be advantageous because they permit slower release over days to weeks. VLPs and rigid inorganic scaffolds are often selected when dense repetitive antigen presentation is needed to increase B-cell receptor clustering or to co-localize antigens and adjuvants in a highly ordered architecture. Therefore, platform selection should be viewed as a cargo- and mechanism-driven design decision rather than a generic formulation choice. Specific examples further clarify this principle. Ionizable lipids such as SM-102-like or MC3-related chemistries have been widely used in mRNA-loaded cancer vaccine LNPs to support nucleic acid encapsulation and pH-triggered endosomal escape [8,15]. PLGA and PLA systems have been used for peptide and protein cancer vaccines because they offer degradable matrices and sustained release, while chitosan-based systems add mucoadhesive and cationic properties that can improve APC uptake [13]. Among inorganic systems, gold nanoparticles and mesoporous silica have been used as antigen-bearing scaffolds or adjuvant co-carriers, while VLPs remain attractive for highly immunogenic repetitive antigen display [14,16].

Nanoparticle size is another critical design variable that should be stated more explicitly. For lymphatic drainage after subcutaneous or intradermal administration, particles in the approximate range of 10 to 100 nm are generally considered optimal, because they can access lymphatic capillaries while still being efficiently retained and sampled in draining lymph nodes. Particles substantially smaller than about 10 nm may diffuse rapidly into blood capillaries or undergo renal clearance, whereas particles larger than about 150 to 200 nm are more likely to remain at the injection site or be taken up locally by phagocytes before effective lymphatic transport. In practice, many successful vaccine nanocarriers fall within the 20 to 100 nm range, although the ideal size also depends on charge, deformability, and route of administration [7].

This combination of safety and immunogenicity has led to the evaluation of VLPs as carriers for tumour-associated antigens in cancer vaccine development [16,17]. Collectively, these nanoparticle platforms provide complementary advantages for cancer vaccine delivery. Selection among them is guided by payload type, desired immunological outcome, and translational considerations, and hybrid systems that integrate features of multiple platforms are also gaining attention to further optimize immunogenicity and clinical performance. A comparative summary of the major nanoparticle platforms used in cancer vaccine development is presented in Table 1. This table summarizes major nanoparticle-based delivery systems, including lipid nanoparticles, polymeric nanoparticles, inorganic nanoparticles, and virus-like particles. Key features such as composition, payload type, advantages, and limitations are highlighted to provide a comparative understanding of their roles in enhancing antigen delivery and immune activation in cancer vaccines.

## 3. Translational Barriers

### 3.1. Tumour Microenvironment Barriers

A major reason why cancer vaccines underperform in solid tumours is the profoundly immunosuppressive tumour microenvironment (TME). Even when nanovaccines improve antigen presentation and dendritic-cell uptake, vaccine-induced effector T cells must still function within a milieu enriched with regulatory T cells, myeloid-derived suppressor cells, M2-like macrophages, inhibitory cytokines such as transforming growth factor-β and interleukin-10, and metabolically hostile features, including hypoxia, acidity, and nutrient deprivation. These factors collectively impair antigen presentation, reduce T-cell infiltration, promote T-cell exhaustion, and favour tumour immune escape. Hypoxia is particularly problematic because it not only restricts cytotoxic lymphocyte activity but also supports immunosuppressive stromal and myeloid programs, thereby weakening the downstream effects of vaccination. As a result, successful nanovaccine design requires more than efficient delivery; it must also account for the dynamic ecological constraints imposed by the TME [18,19]. Another translational challenge is that antigen-specific immune responses generated in peripheral lymphoid organs do not necessarily translate into durable intratumoural control. Tumours often exhibit defective antigen processing, heterogeneous antigen expression, low baseline T-cell infiltration, and adaptive upregulation of inhibitory pathways such as PD-1/PD-L1 and CTLA-4-associated signaling. This means that even potent nanovaccine formulations may fail clinically if they cannot overcome immune exclusion or reverse functional suppression within tumour tissue. Current evidence, therefore, supports the view that TME remodeling is not ancillary but central to the clinical success of nanotechnology-based cancer vaccines [5,6]. To address these barriers, nanovaccines are increasingly being engineered not simply as antigen carriers but as TME-modulating systems. One strategy is to co-deliver immune agonists, such as Toll-like receptor ligands or STING agonists, together with tumour antigens in the same nanoparticle so that dendritic-cell activation and antigen presentation occur in a synchronized fashion. Another is to incorporate hypoxia-responsive, pH-responsive, or redox-responsive materials that release payload preferentially within the tumour milieu. Oxygen-generating or oxygen-carrying nanomaterials have also been explored to partially reverse hypoxia and improve T-cell function, while membrane-coated and biomimetic nanoparticles are being used to improve trafficking into immune-excluded tumours [6,18]. Thus, one of the most important shifts in current nanovaccine design is the movement from passive delivery toward active immunological reprogramming of the tumour microenvironment.

### 3.2. Delivery and Targeting Challenges

Although nanoparticles can protect antigens and adjuvants from premature degradation, in vivo delivery remains a major concern. Following administration, nanovaccines must avoid rapid clearance, drain effectively to lymphatics, be retained in lymph nodes, and reach dendritic cells in a form that supports cross-presentation. Small deviations in particle size, charge, rigidity, hydrophobicity, or surface chemistry can markedly alter biodistribution and uptake. Nanoparticles that are too large may remain trapped at the injection site, whereas those that are too small may be rapidly cleared or distributed non-specifically. In addition, protein corona formation in vivo can mask targeting ligands and change the biological identity of the formulation, reducing reproducibility between experimental systems and clinical settings [7,8,20]. A related issue is that efficient lymph-node targeting does not automatically ensure efficient tumour targeting. For therapeutic cancer vaccines, delivery must be optimized across two spatially distinct compartments: secondary lymphoid tissue, where priming occurs, and the tumour site, where effector function is required. This creates a design trade-off between formulations optimized for immune priming and those optimized for deep tumour penetration or local immune modulation. These competing demands help explain why many promising preclinical nanovaccines lose efficacy during translation to larger animal models and humans, where lymphatic anatomy, immune composition, and pharmacokinetics are more complex [5,7,20]. Researchers are actively engineering nanovaccines to overcome these delivery barriers. Surface decoration with mannose, antibodies, or APC-binding ligands is being used to increase dendritic-cell uptake. Albumin-hitchhiking designs and lymph-node-homing amphiphiles have been developed to improve drainage to secondary lymphoid tissues. pH-responsive and charge-reversal systems are being explored to improve cellular internalization after lymph-node arrival. Furthermore, microfluidic manufacturing now allows tighter control over particle size and polydispersity, which is particularly important for keeping formulations within the size range that favours lymphatic drainage while preserving payload integrity [7,15,20].

### 3.3. Safety and Toxicity Concerns

Safety remains a critical concern because the same physicochemical features that enhance immune activation can also provoke unwanted inflammatory or off-target effects. Cationic and ionizable materials may induce complement activation, infusion reactions, oxidative stress, or local tissue injury, while some inorganic platforms raise concerns regarding persistence, long-term accumulation, and poorly defined degradation products. In immune-oncology applications, excessive innate activation can be counterproductive, leading to systemic reactogenicity without proportional gains in tumour-specific immunity. Furthermore, repeated dosing, which is often necessary for therapeutic cancer vaccination, emphasizes the importance of understanding biodistribution, clearance, immunogenicity of carrier materials, and chronic exposure risks [21]. Safety evaluation is further complicated by the multifunctional nature of modern nanovaccines. Many formulations combine antigens, adjuvants, targeting ligands, membrane coatings, or stimulus-responsive components, each of which can alter pharmacology and toxicity. Regulatory agencies have therefore emphasized extensive chemistry, manufacturing, and controls characterization, along with pharmacokinetic, bioanalytical, and nonclinical safety assessment for complex nanosystems. For translational success, biodegradability, batch-to-batch consistency, and a clearly defined structure-function relationship are increasingly viewed as essential rather than optional design attributes [6]. Current engineering responses to safety concerns include the replacement of permanently cationic lipids with ionizable lipids that become protonated mainly in acidic intracellular compartments, the increasing use of biodegradable polymer backbones and cleavable linkers, and the adoption of lower-immunogenicity helper lipids and excipients. Rational dose optimization, route-of-administration studies, and biodistribution profiling are also becoming central to preclinical development, particularly for formulations intended for repeated therapeutic dosing. These strategies do not eliminate toxicity concerns, but they improve the therapeutic window and make nanovaccine performance more predictable across different tumour contexts.

### 3.4. Manufacturing and Regulatory Issues

Manufacturing remains one of the least discussed, yet most decisive barriers to clinical translation. Many nanovaccine platforms are formulated through multistep processes involving encapsulation efficiency optimization, surface functionalization, sterile processing, purification, and cold-chain stabilization. Minor changes in mixing conditions, excipient composition, solvent removal, or storage can alter particle size distribution, polydispersity, antigen loading, and release kinetics, thereby affecting biological performance. These issues are particularly important for personalized platforms, where the need for rapid lot-specific manufacturing can strain conventional quality control frameworks [5]. Regulatory evaluation is likewise challenging because nanovaccines often do not fit neatly into traditional categories of biologics, drug products, or combination products. Authorities such as the FDA and EMA already require detailed characterization for liposomal and other nano-enabled medicinal products, but platform heterogeneity continues to complicate comparability, scale-up validation, and extrapolation across products. In practice, the translational path is slowed by the need to define critical quality attributes, demonstrate reproducibility across manufacturing lots, and align analytical assays with clinically meaningful product specifications. For cancer nanovaccines to advance more efficiently, platform standardization and early regulatory engagement will be as important as immunologic innovation [22]. The major translational barriers and corresponding emerging strategies to address these challenges are summarized in Table 2, highlighting the major challenges limiting the clinical translation of nanovaccines, including tumour microenvironment-mediated immune suppression, delivery inefficiencies, safety concerns, and manufacturing constraints. Corresponding emerging strategies, such as personalized nanovaccines, mRNA-based platforms, and combination immunotherapy approaches, are highlighted as potential solutions to improve therapeutic efficacy and clinical applicability.

## 4. Emerging Strategies

### 4.1. Personalized Nanovaccines

Personalized nanovaccines are emerging as one of the most promising solutions to the limited specificity of conventional cancer vaccines. By incorporating patient-specific neoantigens derived from tumour sequencing, these platforms aim to focus immune responses on truly tumour-restricted targets while minimizing central tolerance and off-target effects associated with shared tumour-associated antigens. Nanocarriers are especially attractive in this setting because they can co-deliver multiple neo-antigens together with adjuvants, protect labile nucleic acid or peptide cargo, and tune intracellular trafficking to favor cross-presentation. Recent work has emphasized that personalized nanovaccines may be particularly relevant for metastatic disease, where spatial and clonal heterogeneity necessitate multi-epitope, adaptable vaccine formulations [9,16,25,29]. Importantly, the clinical field is beginning to validate the broader concept of personalized vaccines. In high-risk melanoma, the randomized phase 2b KEYNOTE-942 trial showed that individualized neoantigen therapy mRNA-4157 (V940) combined with pembrolizumab improved recurrence-free survival compared with pembrolizumab alone, while maintaining a manageable safety profile [30]. Although this product is not simply a generic “nanovaccine” study, it strongly supports the translational premise that personalized antigen selection, when paired with a scalable nucleic-acid delivery system, can generate clinically meaningful benefit. Such findings are highly relevant to personalized nanovaccine development, especially for LNP-enabled platforms [31]. The concept has also gained support from primary translational studies in other tumours. In pancreatic ductal adenocarcinoma, individualized RNA neoantigen vaccines have been shown to induce measurable neoantigen-specific T-cell responses, supporting the feasibility of this approach even in tumours traditionally considered immunologically “cold” [32]. These data are important because they suggest that personalized nanovaccine strategies may be useful not only in highly mutated tumours such as melanoma but also in lower-mutation-burden malignancies when antigen prioritization and delivery are sufficiently optimized.

### 4.2. mRNA Nanovaccine Platforms

mRNA nanovaccine platforms are gaining momentum because they offer speed, modularity, and manufacturing flexibility that are difficult to achieve with protein- or peptide-based vaccines. Once a tumour antigen or neoantigen set is identified, mRNA constructs can be synthesized rapidly and encapsulated into lipid nanoparticles or related carriers that protect the cargo, facilitate endosomal escape, and promote expression in antigen-presenting cells. This platform also allows multiplexing, enabling simultaneous encoding of several neoantigens or immunostimulatory factors within one formulation. The success of LNP-mRNA technologies in infectious disease has accelerated their adoption in oncology and strengthened confidence in their scale-up potential [15]. For cancer applications, mRNA nanovaccines are especially attractive because they integrate personalized antigen design with a delivery platform already supported by robust pharmaceutical development experience. Their remaining limitations, including innate immune sensing, stability, endosomal escape efficiency, and organ-selective biodistribution, are now active engineering targets rather than conceptual obstacles. As ionizable lipid chemistry, microfluidic formulation methods, and payload optimization continue to improve, mRNA nanovaccines are increasingly positioned as a leading translational format for therapeutic cancer vaccination [33].

More specifically, several technological innovations are being pursued to improve endosomal escape, one of the major bottlenecks in mRNA delivery. These include the design of ionizable lipids with optimized pKa values that become protonated in acidic endosomes, the incorporation of helper lipids with fusogenic behavior, the use of pH-responsive or membrane-destabilizing polymers, and the tuning of nanoparticle internal architecture to promote non-bilayer phases associated with membrane disruption. Microfluidic formulation methods are also being used to improve the reproducibility of LNP structure, which in turn influences cytosolic release efficiency. Thus, endosomal escape is increasingly being addressed through structure-activity-guided engineering rather than empirical formulation alone [8,15,33,34].

### 4.3. Combination with Immune Checkpoint Therapy

**Figure 2:**
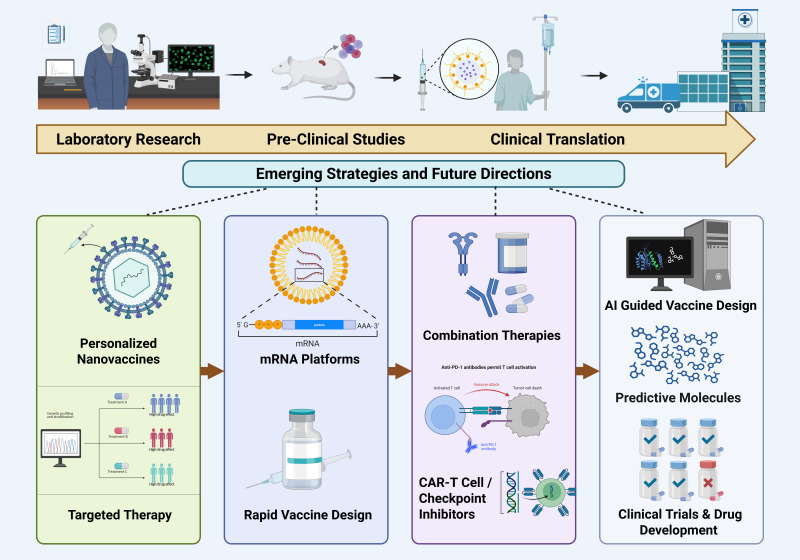
Emerging strategies and future directions in nanotechnology-based cancer vaccines. Recent advances in nanotechnology are driving the development of next-generation cancer vaccines through multiple innovative strategies. Personalized nanovaccines enable patient-specific antigen targeting, improving therapeutic precision. mRNA-based platforms facilitate rapid vaccine design and scalable manufacturing. Combination of immunotherapies, including CAR-T cell therapy and immune checkpoint inhibitors, enhance antitumour immune responses when integrated with nanovaccine approaches. Artificial intelligence (AI)-guided vaccine design supports predictive antigen identification and optimization of vaccine candidates. These strategies collectively contribute to the translational pathway from laboratory research and pre-clinical studies to clinical development and therapeutic application in cancer immunotherapy. These findings reinforce the view that the future of nanovaccine translation will involve rational combination regimens rather than stand-alone vaccine products.

A strong emerging strategy is to combine nanovaccines with immune checkpoint blockade. Conceptually, this is highly rational: vaccines expand tumour-specific T-cell clones, while checkpoint inhibitors rescue or sustain their function within the tumour microenvironment. This combination addresses one of the major weaknesses of vaccine monotherapy, namely that immune priming alone is often insufficient in the face of established intratumoural suppression. Preclinical and translational reviews consistently show that vaccine-checkpoint combinations can increase T-cell infiltration, improve memory formation, and shift the balance of the TME toward effective antitumour immunity [35]. Clinical evidence is also beginning to support this strategy beyond melanoma. In advanced hepatocellular carcinoma, a phase 1/2 study showed that a personalized neoantigen vaccine combined with pembrolizumab induced new neoantigen-specific T-cell responses and demonstrated clinical activity, supporting the idea that vaccination can complement PD-1 blockade by generating immune responses that checkpoint inhibition alone may not initiate [36]. Emerging approaches to overcome current translational barriers in nanovaccine development are summarized in Figure 2, which highlights strategies such as personalized nanovaccines, mRNA platforms, combination immunotherapy, and AI-guided vaccine design.

## 5. Future Perspectives

### 5.1. Artificial Intelligence (AI)-Guided Vaccine Design

Artificial intelligence (AI) is likely to become an important enabling technology for next-generation cancer nanovaccines. One of the major concerns in personalized vaccine development is the accurate identification of immunogenic neoantigens from a large background of passenger mutations. AI and machine-learning models are now being applied to integrate peptide-MHC binding prediction, antigen processing, transcript expression, clonality, and even T-cell receptor recognition, thereby improving prioritization of candidate neoepitopes. Beyond target selection, AI is increasingly being used to optimize codon usage, untranslated regions, mRNA structural features, and formulation parameters that influence expression and stability [37,38]. The role of AI can also be expanded from antigen selection to nanoparticle design itself. Data-driven models are increasingly being explored to predict how changes in lipid tail structure, ionizable head groups, PEG-lipid content, particle size, and mixing conditions influence encapsulation efficiency, stability, organ tropism, and endosomal escape. In principle, this could reduce empirical screening burdens and accelerate the identification of formulation windows most likely to yield clinically useful delivery behaviour. AI-guided design is therefore relevant not only to “what antigen should be encoded” but also to “what carrier architecture is most likely to deliver it effectively.” In the future, AI-guided workflows could shorten the time from tumour biopsy to individualized nanovaccine manufacture, which is essential for aggressive cancers. However, the value of these systems will depend on access to large, well-annotated immunopeptidomics and clinical datasets, as well as transparent validation across tumour types and patient populations. Thus, AI should be framed not as a replacement for experimental immunology but as a force multiplier that can accelerate design, reduce attrition, and support more rational nanovaccine development [38].

### 5.2. Next-Generation Nanocarriers

The next wave of nanovaccine innovation will likely come from smarter carrier systems rather than simply new payloads. Biomimetic nanoparticles, membrane-coated carriers, self-assembling protein systems, and stimulus-responsive materials are being developed to improve immune-cell targeting, reduce off-target clearance, and respond to local cues such as pH, redox state, or hypoxia. Such systems may help bridge the current gap between efficient lymph-node priming and effective tumour-site activity. In parallel, multifunctional carriers that integrate antigen delivery with adjuvant release, TME remodeling, or imaging capability may support more precise and personalized immunotherapy [39]. A particularly important direction is the development of nanocarriers that balance complexity with manufacturability. The most clinically successful future platforms are unlikely to be the most elaborate ones, but rather those that combine strong biological performance with reproducible synthesis, scalable formulation, and tractable regulatory characterization. In this respect, carrier engineering must increasingly be guided by translational criteria from the outset, including stability, sterility, analytical tractability, and compatibility with rapid personalized manufacturing workflows [40].

### 5.3. Clinical Translation Outlook

The clinical translation outlook for nanotechnology-based cancer vaccines is more promising now than at any point in the past, but progress will likely be incremental and indication-specific. Early clinical success with personalized neoantigen vaccines and the broader validation of LNP-enabled nucleic-acid delivery have strengthened the field’s translational foundation. Even so, meaningful clinical impact will depend on selecting the right disease settings, such as minimal residual disease, adjuvant therapy after resection, or immunologically responsive tumours, where vaccine-induced T cells have the greatest chance of altering outcome [41]. Looking ahead, the most effective nanovaccine strategies will probably combine four elements: precise antigen selection, clinically scalable carriers, rational combination therapy, and biomarker-guided patient stratification. Rather than asking whether nanovaccines will replace other immunotherapies, the more realistic question is how they can be integrated into multimodal treatment algorithms. If advances in AI-assisted design, carrier engineering, and regulatory standardization continue at their current pace, nanotechnology-based cancer vaccines could move from experimental adjuncts to a practical component of precision oncology [42].

## 6. Conclusions

Nanotechnology-based cancer vaccines have emerged as a promising strategy to enhance tumour-specific immune responses by improving antigen delivery, protection, and immune activation. Advances in nanoparticle platforms, including lipid, polymeric, inorganic, and virus-like particle systems, have enabled the development of more efficient and adaptable vaccine formulations that overcome several limitations of conventional cancer vaccines. Despite these advances, major translational challenges remain, including immunosuppressive tumour microenvironments, inefficient intracellular delivery and antigen presentation, safety concerns, manufacturing complexity, and regulatory barriers.

Emerging approaches such as personalized nanovaccines, mRNA-based vaccine platforms, combination immunotherapy, and artificial intelligence-guided vaccine design are rapidly reshaping the field and offer new opportunities to improve therapeutic efficacy and clinical applicability. Importantly, the future success of cancer nanovaccines will depend not only on immunological potency but also on the development of clinically scalable, reproducible, and safe delivery systems.

Overall, continued integration of nanotechnology, cancer immunology, biomaterials engineering, and precision medicine is expected to accelerate the translation of nanovaccine platforms from experimental research into clinically effective cancer immunotherapies.

## Figures and Tables

**Table 1:** Overview of nanoparticle platforms used in cancer vaccine development.

Platform	Material	Payload Type	Cancer Type (Example)	Size (nm)	Advantages	Limitations	Reference
Lipid nanoparticles	Ionizable lipids, cholesterol, PEG-lipids	mRNA, protein, peptide	Melanoma, solid tumours	50–150	High biocompatibility, lymph node targeting, scalable	Stability, potential immune reactogenicity	[8]
Polymeric nanoparticles	PLGA, PLA, chitosan	Peptides, proteins, and DNA	Multiple solid tumours	50–300	Biodegradable, tunable release, surface functionalization	Manufacturing complexity, batch variability	[13]
Inorganic nanoparticles	Gold, silica, MOFs	Peptides, proteins	Multiple cancers (preclinical models)	10–100	High antigen loading, imaging/theranostics	Long-term accumulation, toxicity concerns	[14]
VLPs	Self-assembling proteins	Peptides, proteins	HPV-associated cancers (e.g., cervical cancer)	20–200	Highly immunogenic, safe, multivalent display	Limited payload flexibility, production complexity	[17]

**Table 2:** Key translational barriers in nanotechnology-based cancer vaccines and emerging strategies to overcome them.

Barrier	Description	Impact on Vaccine Efficacy	Emerging Solutions	Reference
Tumour microenvironment	Immunosuppressive cytokines, Tregs, and hypoxia	Reduced T-cell activation, immune evasion	Combination with checkpoint inhibitors, tumour-targeted delivery	[23]
Delivery challenges	Poor lymph node trafficking, rapid clearance, physicochemical instability, inefficient intracellular delivery, and suboptimal antigen presentation	Reduced antigen availability and weak immune activation	Size/charge optimization, LNPs, targeting ligands, controlled release systems	[8,24]
Antigen presentation & intracellular trafficking	Inefficient endosomal escape, limited cross-presentation	Poor CD8^+^ T-cell activation	Endosomal escape strategies, ionizable lipids, antigen engineering	[25]
Adjuvant co-delivery	Inadequate co-localization of antigen and adjuvant	Weak dendritic cell activation	Co-encapsulation strategies, TLR/STING agonists	[26]
Route of administration	Variability in immune activation based on delivery route	Inconsistent immune responses	Route optimization, depot-forming systems	[27]
Safety & toxicity	Off-target immune activation, nanoparticle accumulation	Limits dosing and clinical use	Biodegradable polymers, optimized dosing	[13,28]
Manufacturing & regulatory	Complex synthesis, reproducibility issues	Delayed clinical translation	Scalable LNP/mRNA platforms, standardized protocols	[6]

## Data Availability

No new data was generated during the preparation of the manuscript.
